# Electron tunneling between vibrating atoms in a copper nano-filament

**DOI:** 10.1038/s41598-021-86603-6

**Published:** 2021-04-01

**Authors:** Mohammad Al-Mamun, Marius Orlowski

**Affiliations:** Bradley Department of Electrical and Computer Engineering, Virginia Tech, Blacksburg, VA 24061 USA

**Keywords:** Thermoelectric devices and materials, Quantum information, Nanoscience and technology

## Abstract

Nanowires, atomic point contacts, and chains of atoms are one-dimensional nanostructures, which display size-dependent quantum effects in electrical and thermal conductivity. In this work a Cu nanofilament of a defined resistance and formed between a Cu and Pt electrode is heated remotely in a controlled way. Depending on the robustness of the conductive filament and the amount of heat transferred several resistance-changing effects are observed. In case of sufficiently fragile nanofilament exhibiting electrical quantum conductance effects and moderate heating applied to it, a dramatic increase of resistance is observed just after the completion of the heating cycle. However, when the filament is allowed to cool off, a spontaneous restoration of the originally set resistance of the filament is observed within less than couple tens of seconds. When the filament is sufficiently fragile or the heating too excessive, the filament is permanently ruptured, resulting in a high resistance of the cell. In contrast, for robust, low resistance filaments, the remote heating does not affect the resistance. The spontaneous restoration of the initial resistance value is explained by electron tunneling between neighboring vibrating Cu atoms. As the vibrations of the Cu atoms subside during the cooling off period, the electron tunneling between the Cu atoms becomes more likely. At elevated temperatures, the average tunneling distance increases, leading to a sharp decrease of the tunneling probability and, consequently, to a sharp increase in transient resistance.

## Introduction

Recently metallic nanofilaments have attracted a great deal of attention due to its interesting physics and their great potentials for applications like neuromorphic computing, resistive non-volatlie memory, flexible touch screen, transparent electrodes and solar cells and transparent electrodes^1–7^. For the design and optimization of these devices, the electrical and thermal properties of an individual nanofilament are both fundamental and critical. Up to now, the electrical quantum conduction properties of nanofilaments and nanocontacts have been widely reported^8–10^. However, characterization of thermal conduction properties is experimentally more difficult to measure. Accordingly the literature on this topic is rather scarce^11–15^ there in. Mosso et al.^12^ established the validity of the Wiedemann–Franz law down to atomistic dimensions showing that the quantum thermal conductance G_th_ is proportional to the electric quantum conductance G_o_ = 2e^2^/h even for atomic contacts, G_th_ = L_o_ × T × G_o_, where e is the elementary electronic charge, h the Planck constant, L_o_ the Lorentz number, and T the absolute temperature in Kelvin. In this work, we investigate the electric conductivity of remotely heated Cu nanofilaments in a resistive Cu/TaO_x_/Pt memory cells.

Resistive random access memory (ReRAM) is one of the prime candidates for the nonvolatile memory^16–27^ supplanting the current floating gate technology because of its excellent scalability, simple metal–insulator-metal (MIM) structure, low fabrication cost, 3D integration feasibility, and promising performances in power consumption, speed, retention and endurance. ReRAM stores information (bits) based on the resistive switching effect. Under appropriate external electrical field, the resistance state of the ReRAM device can be reversibly switched between a low resistance state (LRS) or ON-state and high resistance state (HRS) or OFF-state. The resistive switching can be a localized or a uniform phenomenon. In so-called phase change memory materials, uniform switching scales proportionally with the total volume of the switching material of the MIM structure, while localized switching is realized with the formation and rupturing of a conductive filament (CF). Filamentary ReRAM memory cell serves as a convenient platform for studying the various physico-chemical effects. In the filamentary type ReRAM cell, when the nanofilament in the insulating layer is formed, ReRAM changes to ON-state. If the nanofialment is ruptured, the device switches back to OFF-state. The formation and rupture of the nanofilament has been explained by migration of metal cation or oxide defect anion under applied bias enabled by electrochemical redox reactions of the metal ions or oxide point defects such as oxygen vacancies. The length of the nanofialment in a ReRAM cell is determined by the thickness of the dielectric layer while its lateral dimension can be electrically modulated and are in the order of a few to several tens of nanometers as evidenced by the observation of atomic force microscopy (AFM)^28–30^, high-resolution transmission electron microscopy (HRTEM)^31–44^, and scanning TEM (STEM)^45^. As the CF size is in the range of nanoscale to atomic size, which is comparable to the mean free path or the Fermi wavelength of the conduction electron, the scattering of electrons might be absent, resulting in ballistic electron transport^46,47^ and the quantized conductance^48–50^. In recent studies, conductance quantization phenomena have been demonstrated to be present in the nanofilaments in ReRAM structures^51–54^. Hence, it is important to understand the interplay of electrical and thermal effects in a nanofilaments in order to fine-tune the performance, assess the reliability, and control the variability^55,56^ of ReRAM memory arrays or of neuromorphic devices used in deep machine learning^1^.

## Experimental

**Figure 1:**
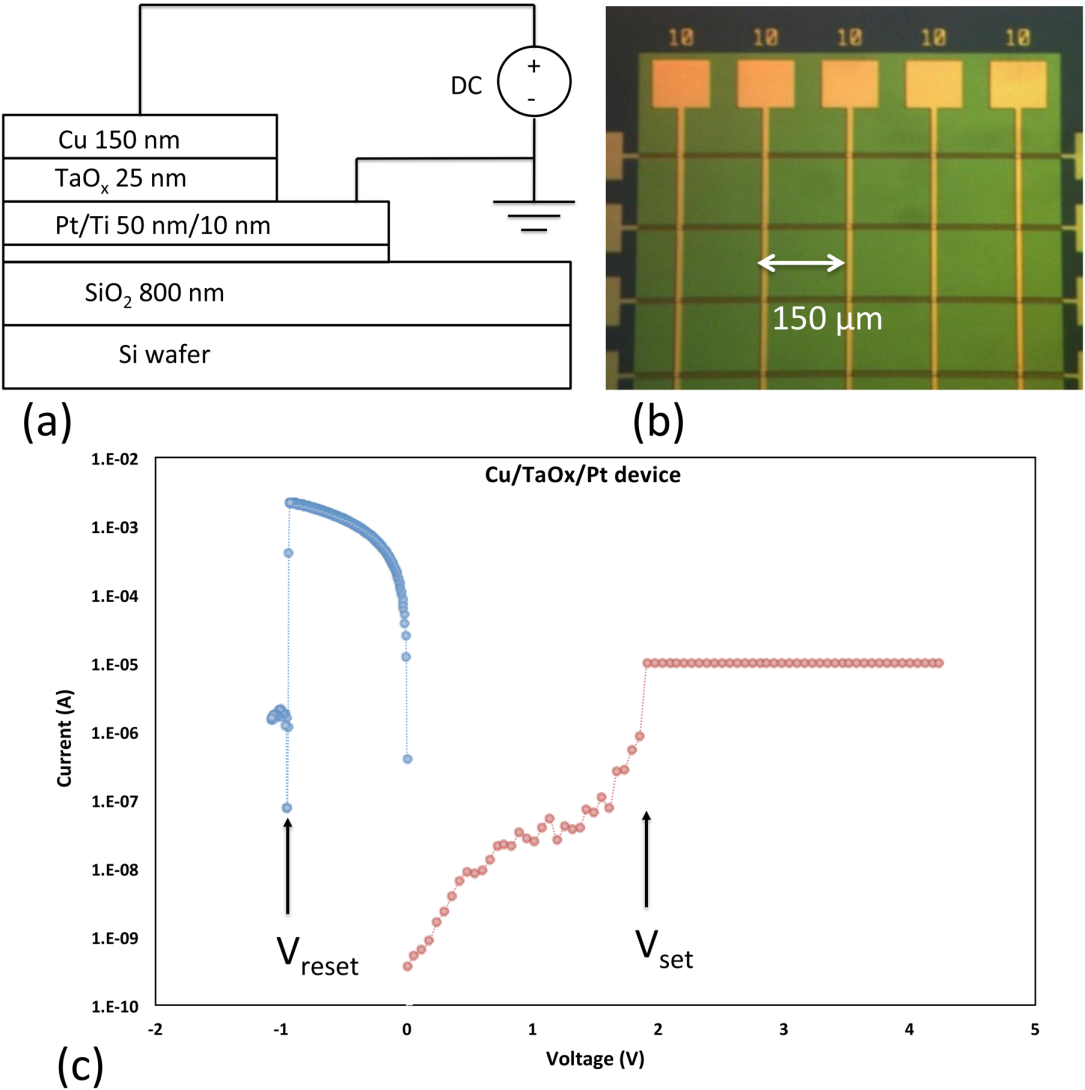
(**a**) Cross-sectional view of a Cu/TaO_x_/Pt resistive switching cell with layer thicknesses indicated. (**b**) Optical microscope picture of an array of Cu and Pt lines with Cu/TaO_x_/Pt cells at the intersections. (**c**) Typical switching cycle with set and reset operations with the threshold voltages V_set_ and V_reset_ respectively.

Most commonly, ReRAM cells lie at the intersection of perpendicular metal electrode lines in a crossbar array as shown in Fig. 1b. The Cu/TaO_x_/Pt/Ti resistive RAM cell arrays (Fig. 1b) have been fabricated in a crossbar array on a thermally oxidized Si wafer^57^ with a SiO_2_ layer 650 nm thick. Cu (150 nm), Pt (50 nm), Ti (25 nm) have been deposited by e-beam PVD. The thickness of the electrodes and of the switching layer is shown in Fig. 1a. The Pt electrode lines are patterned with lift-off technique with photoresist thickness of 2 μm to make sure that the sidewalls are sloped gently. The oxygen-deficient TaO_x_ of 25 nm was deposited in blanket fashion by evaporating TaO_x_ pellets without O_2_ injection into the PVD evaporation chamber. The thickness of the TaO_x_ on the sloped sidewalls of Pt line is 98% of the planar thickness as confirmed by Ta_2_O_5_ atomic layer cells manufactured with Ta_2_O_5_ deposition by atomic layer deposition (ALD)^58^. The width of the metal lines varies between 1 μm and 35 μm. The distance between neighboring electrode lines is 150 μm. The memory cells used in this investigation were described in much detail in^58,59^ and in the supplementary file. The electrical characterization was performed at 300 K on a probe station equipped with Keithley 4200-SCS. Before a measurement on a memory cell is taken two grounded needles are placed on the cell contacts for at least 20 s to make sure that the cell capacitor is fully discharged. Then the voltage of the Cu electrode starting at 0 V is ramped at a ramp rate, *rr*, between 0.01 V/s and 2 V/s. During the set operation, a compliance current, I_cc_ of 5 μA to 1 mA has been imposed without an off-chip resistance lest the device be permanently damaged.

When a positive voltage is applied to the Cu electrode, Cu cations are generated according to the redox reaction:

$$Cu \leftrightarrow Cu^{ + } + \, e^{ - } \left( {\text{Reduction}} - {\text{Oxidation}} \right)$$

and migrate under the influence of the electric field in the solid electrolyte to be electrochemically reduced on the surface of the Pt cathode which acts as an effective diffusion barrier for Cu atoms. As more and more Cu atoms accumulate, the copper atom protrusion grows and a metallic filament (CF) of Cu atoms as building blocks forms a conductive path between two electrodes. An abrupt onset of conductance occurs at a threshold voltage V_set_. The filament can be partially undone by applying a negative voltage to the Cu electrode. The abrupt rupturing of the filament manifest itself in the I–V characteristics by a threshold voltage V_reset_. A typical I–V characteristic for set and rest operations is shown in Fig. 1c.

Switching of a cell repeatedly leads to an accumulation of depositions of Joules heat in the device. It has been recently shown that the Joules heat is transported along the shared electrode metal lines affects the neighboring cells and causes the deterioration of their electrical properties^60^. In this work we use the resistive switching memory array to characterize the electrical conductivity of a Cu nanofilament formed in a resistive switching memory cell when heated remotely by a neighboring memory cell along one of the common metal electrodes. The heat generated in the heated memory cell can be controlled by the voltage ramp applied during the reset operation and by the number of consecutive set-reset cycles.

### Remote heating of Cu filament in a Cu/TaO_x_/Pt cell

As described in more detail in^6,61^ a frequent switching of a cell leads to a deposition of Joules heat in that cell that can spread to neighboring cells disposed along one of the electrodes shared with the heated cell. Even those cells that do not share any of the two electrodes with the heated cell can be affected by the thermal cross-talk provided the intermediate cells are set in the ON-state whereby the Cu filaments provide a thermal conduit for the heat transport. This phenomenon allows remote heating of neighboring cells to a various degree dependent on the amount of heat dissipated in the heated cell, on the selected shared electrode and the distance of the cell from the heated cell.

**Figure 2:**
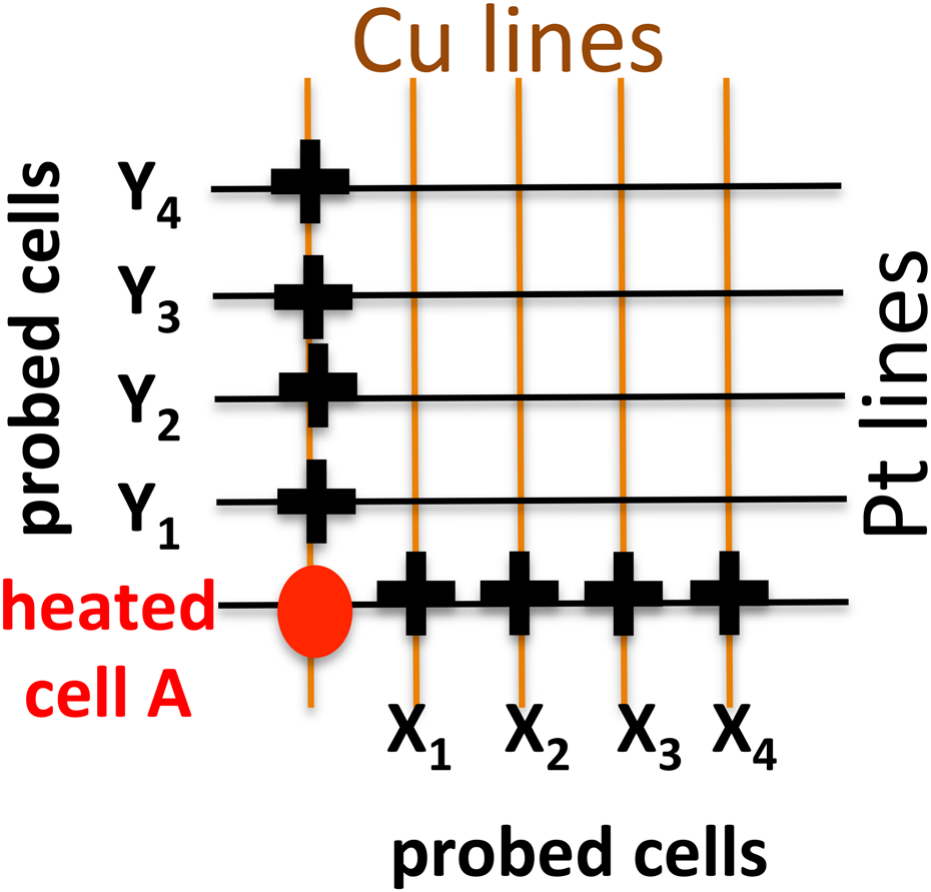
A crossbar array of Cu/TaO_x_/Pt cells. The cell A marked by the red dot represents the heat source cell. The cells affected by the heat source are disposed along the Cu and Pt line and marked by black crosses and X_i_ and Y_j_. All these probed cells are set prior to heating by cell A to an ON-state under various I_cc_ constraints. Immediately after the heating of cell A has ended, the conductivity of the cells X_i_ and Y_j_ is probed at small voltage bias ≤ 0.1 V.

In this work, we are interested in cells that have been set prior to the heating of the neighboring (heated) cell, into a LRS state under various levels of compliance current, I_cc_ and that are connected by a thermal path to the heated device as shown in Fig. 2. It is known that the imposition of I_cc_ during the set operation controls the resistance of the LRS state, R_on_, via the relation^2^

1 $${\text{R}}_{{{\text{on}}}} = {\text{K}}/{\text{I}}^{{\text{n}}}_{{{\text{cc}}}}$$

where K extracted from experimental data for our Cu/TaO_x_/Pt devices yields K ≈ 0.29 V and n ≈ 1. It has been shown^2^ that the constant K for n = 1 represents the minimal voltage under which the device can be set. This has been confirmed following the procedure outlined in^2^ by applying a constant voltage to a reset cell and measuring the current as a function of time. For all voltages in excess of 0.287 V the cell can be set to an ON-state, whereas for voltages smaller than 0.287 V the cell remains in the OFF-state, even when the voltage is applied for a long period of time, in our case, for more than six hours. This result has been verified on more than 75 cells with various width of the Cu and Pt electrode lines. The dependence of R_on_ an I_cc_ allows to form stable but weak, i.e. highly resistive (R_on_ = 50 kΩ), Cu filaments at I_cc_ = 5 μA, and strong, low resistive (R_on_ = 500 Ω) Cu filaments at I_cc_ = 0.2 mA. For compliance current values below 5 μA the filament does not form at all or becomes volatile and ruptures unaided and spontaneously. On the other hand, robust low-resistance Cu filaments formed at I_cc_ > 0.25 mA are prone to become unresettable, i.e. meaning that the cell has been permanently damaged.

## Results

In the context of this work, we are interested in the characterization of those cells set to an ON-state that are neighbors of the heated cell along one of the shared electrode lines as shown in Fig. 2. After setting the cells marked by black crosses to ON-state we heat the device marked by a red dot in Fig. 2. The heating of the cell A can be controlled by a number of sequential switching (set-reset) cycles and by the voltage ramp rate, *rr*, during the reset cycles. To maximize the heating, a low *rr* = 0.1 V/s may be chosen for the reset operation providing a long heating time prior to reaching the threshold voltage V_reset_. For a low *rr*, the current lingers for a long time and hence low *rr* maximizes heating of the cell. For a cell to which a constant voltage ramp rate is applied, the Joules heat Q_JH_ can be calculated by Eq. (2)^62^ where relation between R_on_ and I_cc_ [see Eq. (1)] has been used to obtain the final expression.

2 $${Q}_{JH}={\int }_{0}^{{t}_{res}}\frac{{V}^{2}\left(t\right)}{{R}_{on}}dt ={\int }_{0}^{{V}_{res}/rr}\frac{{rr}^{2}\times {t}^{2}}{{R}_{on}}dt =\frac{{V}_{res}^{3}\times {I}_{cc}}{3\times rr\times K}$$

Here t_res_ is the reset time and is related to V_reset_ via the ramp rate *rr*, t_res_ = V_res_/rr^62,63^. For our devices, one obtains for Q_JH_ according to Eq. (2) ~ 3–5 μJ^63^. The above equation has the advantage that the heat dissipated in the resistive memory cell is described in terms of experimentally measurable parameters, such as, the voltage ramp rate *rr*, and the reset voltage V_res_, and the compliance current I_cc_. It is seen that the heat Q_JH_ is proportional to the inverse of the ramp rate. When the cell is switched repeatedly, say N times on and off in a quick succession, the total heat deposited in the cell will be Q_tot_ = N × Q_JH_ × f, where f is a dimensionless efficiency factor, f < 1, accounting for the heat loss of the device between the switching events. The heat transferred to a neighboring cell, as shown in Fig. 2, is then Q_tran_ = N × Q_JH_ × f × t_tran_, where the coefficient t_tran_ < 1 denotes the efficiency of thermal transfer from the heated cell to neighboring cell (Fig. 2). In Ref.^6^ it has been found that t_trans_ along the Cu line is larger than along the Pt line^61^.

Several thermal models have been put forward in the literature to estimate the temperature distribution in ReRAM cells. Mickel et al.^64^ proposed a geometrically equivalent circuit to calculate the heat transport through the conductive filament, yielding temperature distribution of the memory cell as a function of the spatial dimensions. The critical temperature responsible for the rupturing of a filament has been calculated to be ~ 1225° K. In another work, Mickel et al.^65^ proposed a set of constitutive equations describing the evolution of the heat transport. Based on this approach, the temperature responsible for the rupturing of the filament may be even as high as ~ 1500° K. Sun et al.^66^ have also found that the peak temperature of the filament is somewhere between 600 and 900 °C. Most of the models assumed a thermal boundary condition at the mesoscopic electrodes to be the room temperature. Our results clearly refute this assumption. The electrode lines are heating up considerably and enable the thermal cross-talk between the cells of the array. Karpov et al.^67^ discussed a thermal model for a metallic filament with the temperature boundary condition other than room temperature that is adequate to the thermal cross talk situation. Fangohr et al.^68^ investigated Joule heat in nanowires with various constriction geometries and found that the temperature at the constriction as high as 1336 K. Thus there is a wide consensus that the local peak temperature of the filament can be very high and this temperature is bound to increase further when the cell is switched on and off sequentially with high switching frequency.

**Figure 3:**
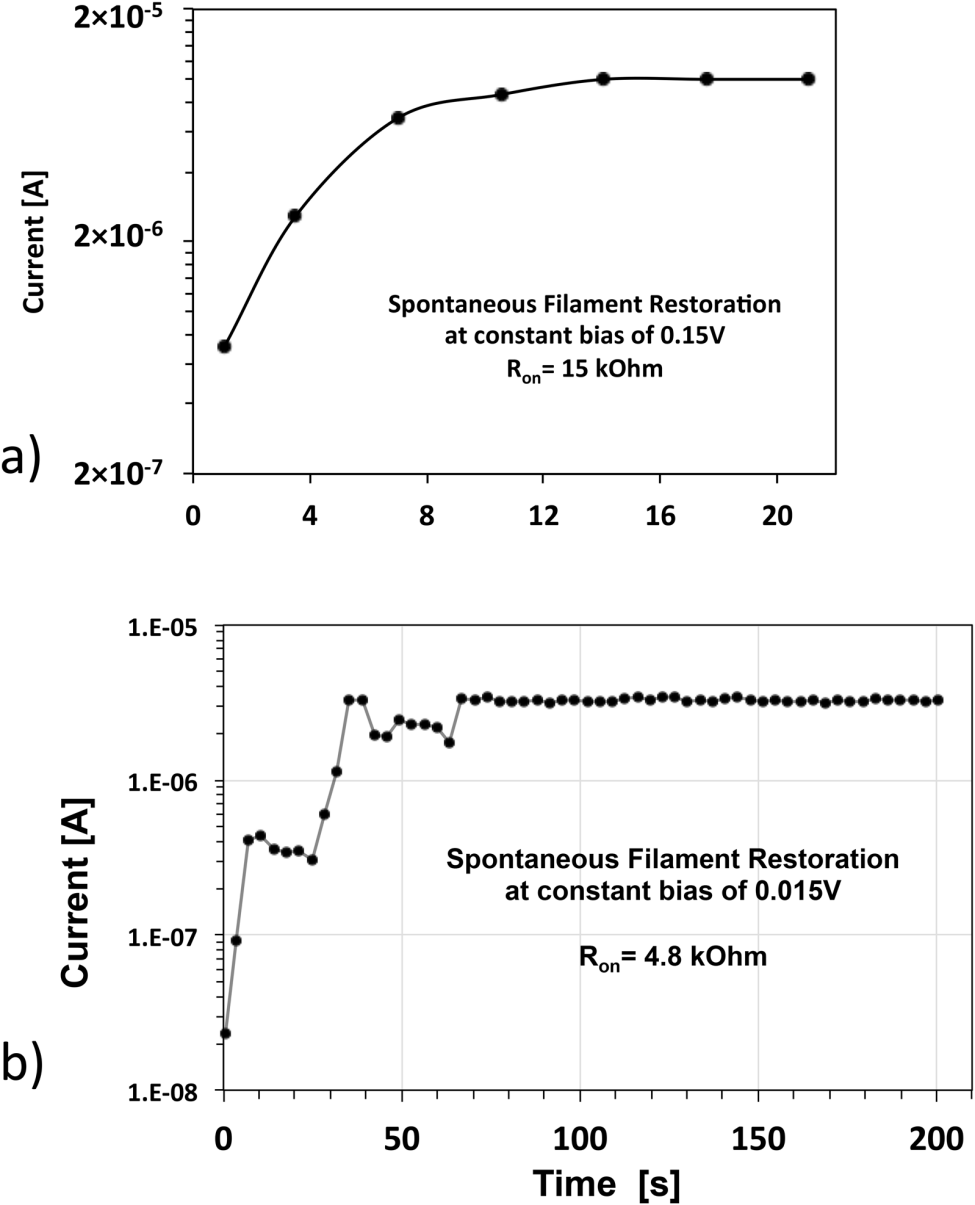
(**a**) Temporal evolution of the current through a cell X_1_ just after the heating of the heart source cell A measured at a constant bias of 0.150 V. The cell X_1_ has been initially preset to an ON-state with R_on_ = 15 kΩ. (**b**) Temporal evolution of the current through a cell Y_1_ just after the heating of the heart source cell A measured at a constant bias of 0.015 V. The cell Y_1_ has been initially preset to an ON-state with Ron = 4.8 kΩ.

The key experiment is conducted in the following way; we test the electric properties of the neighboring cells (marked by black crosses and set to the on-state prior to the experiment in a preset operation) immediately after the heating of the cell A has ended (i.e. after about 50 s needed to reposition the needle on the probe station). The probing of the cell is done at much smaller voltages than 0.288 V, the minimum set voltage to set an off-state cell into a LRS state. As seen below in Fig. 3, the probing voltages are 0.15 V and 0.015 V. We find that in most cases, the neighboring cell has been reset to an OFF-state despite having been set to an LRS state prior to the heating. The ramp rate during the probing is chosen to be high rr = (1–2)V/s to minimize the heating effects of the probed cell caused by the electrical characterization itself. Our key findings fall into three categories:

(i) for filaments formed at high compliance currents, i.e. larger than 0.2 mA, the probed cell remains in an ON-state state with the same resistance R_on_ when the cell was set to the ON-state. In some cases, we find somewhat degraded resistance R_on_ shifted to slightly higher values compared with the original R_on_ value for the preset operation.
(ii) For all probed cells that have been set at I_cc_ < 0.2 mA, we find that the probed cell is highly resistive, i.e. in an OFF-state. However, when cells X_i_ and Y_j_ are probed again, after 250 s or longer, we find the same cell no longer in the OFF-state but spontaneously back to the ON-state with R_on_ in most cases the same as the originally R_on_ set before heating the neighboring cell, and a few cases with slightly higher resistance than the initial R_on_ at the preset operation. Thus, during the heat dissipation period of 250 s or longer, the filaments in the probed cell apparently recover spontaneously and restore themselves to the same R_on_ value to which they have been set in the preset operation.
(iii) In a few cases, we were able to capture the time evolution of the spontaneous restoration of the filament; two instances of such spontaneous restoration are shown in Fig. 3, which provides information on the characteristic timescale of thermal dissipation of the heated and probed cells. For our specific Cu/TaO_x_/Pt array, the time heat dissipation constant appears to be on the order of 10 s. The time constant depends strongly on the thermal conductivity of the metal electrodes^61,69^.

For the current vs time curve at a constant voltage of 0.15 V, as shown in Fig. 3a, the original R_on_ of the cell was set at I_cc_ = 23 μA to yield R_on_ = 15 kΩ. One can see a gradual transition from a current flowing through the cell from 7 × 10^–7^ to 1 × 10^–5^ A over a time period of 22 s. Almost all of the transitions from low current to high current level that we were able to capture are smooth without any distinct features. They all are seen at constant bias larger or equal to 0.15 V. Another instance of a spontaneous restoration is seen in Fig. 3b at a still smaller voltage of 0.015 V. In this case, the cell was set in the preset operation to R_on_ = 4.8 kΩ. It is seen that the current through the filament current recovers over ca. 100 s to a stable value, corresponding to the same resistance R_on_ = 4.8 kΩ established in the preset operation. In this case the spontaneous restoration proceeds in discrete steps and displays even negative resistance values. We were not able to capture more curves like this one. The reason is that the discrete transitions appear to happen within the first 10 s and in most of our attempts to measure it we fail because it takes us 50 s to 70 s to replace the needles of the probe station from heating the device A to probe the device X_1_ or Y_1_. Thus, we begin our measurement when this initial transition period is already over. A better measurement set up will give us access to this interesting transition region at the beginning of the cooling off time. Hence, we conclude that the current at such small voltages is just monitoring and not impacting the spontaneous recovery of the filament’s initial resistance due to the ensuing heat dissipation.

Therefore, in absence of other discoverable factors, we hypothesize that the spontaneous restoration of the filament is caused by the temporal dissipation of the heat along the shared electrode and the concomitant temperature decrease of the Cu filament in the probed cell.

We should note that instances of temporal evolution of the current through the filament under a constant bias, as shown in Fig. 3, are difficult to capture. They depend sensitively on the right amount of remote heating and on the robustness of the filament. If the heating is too low, R_on_ is hardly degraded and the cell persists in the initial ON-state. If the heating is too large, the filament is ruptured, displaying a high resistance R_off_. In the case of strong heating, the vibrations of Cu atoms are so large that the atoms are displaced from their original locations impairing the filament structurally. The heating for the purpose of observing transient effects shown in Fig. 3, is just right when the Cu atoms vibrate with close to maximum amplitude however, without leaving their matrix location. The above described effect of transient restoration of the filament cannot be observed for filaments formed at small I_cc_: 5μA < I_cc_ < 20μA. Neither, could they been observed for cells set at high I_cc_ with 0.2 mA < I_cc_ < 1 mA, no matter how widely the remote heating has been varied. However, for intermediate I_cc_ values, 23 μA < I_cc_ < 150 μA, the effect can be repeatedly, albeit rarely, observed.

**Figure 4:**
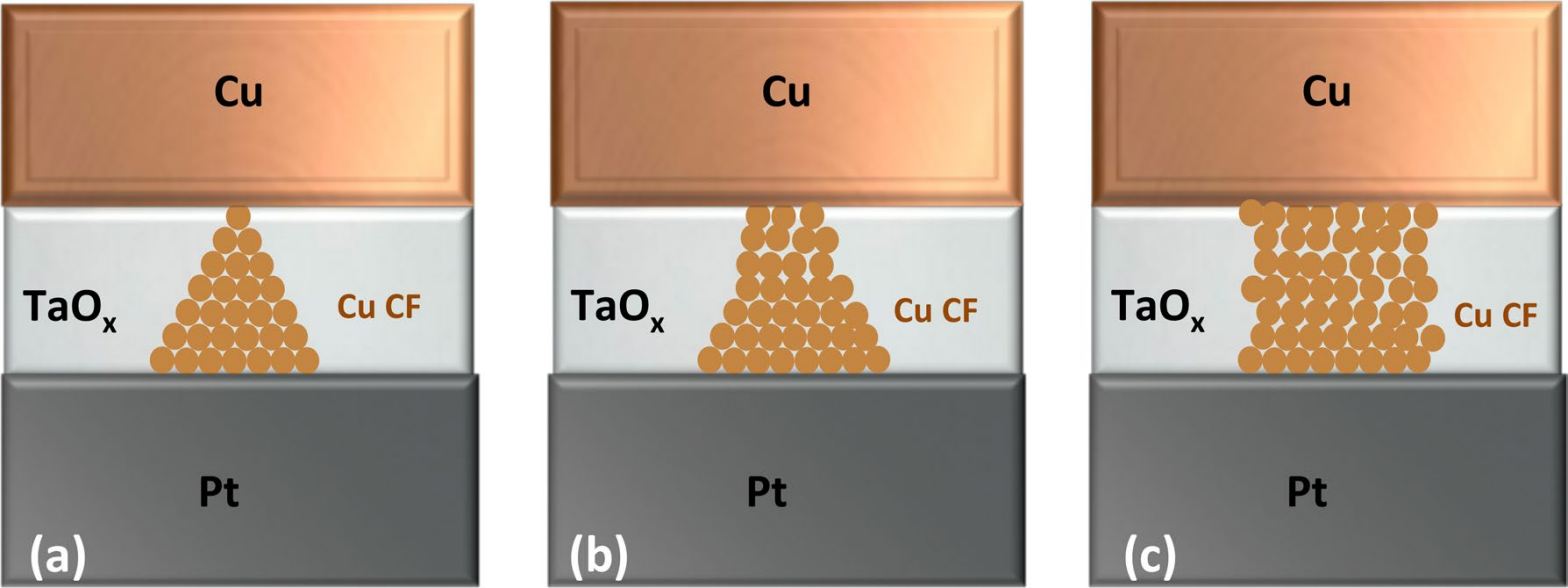
Model geometry for Cu conductive filaments (and also nanocontacts) formed at various I_cc_ levels. (**a**) Filament formed at low I_cc_ resulting in a single atom contact. (**b**) Filament formed at moderate I_cc_ resulting in few atom contact. (**c**) Filament at a high I_cc_ resulting in a cylindrically-shaped filament with mesoscopic contacts with both electrodes.

Hypothesized shapes of the Cu filament for the three I_cc_ regimes are shown Fig. 4 as variations of a truncated cone^6^. For a very weak, i.e. highly resistive filaments a cross-section of the constriction at the top touching the Cu electrode of the truncated cone could contain as few as 1–9 copper surface atoms. The bulk of the resistance resides in the segment of the filament containing the restriction. For medium I_cc_ currents, the constriction at the blunted tip of the cone is still there but consists now of more atoms, e.g. 10–36 atoms. Finally, for the high I_cc_ current levels, the constriction disappears and the shape of the filament assumes a more cylindrical form. Such filaments are difficult to rupture as the locus of the maximum temperature resides in the cylinder half way between the two electrodes.

### Modeling of the filament resistance as a function of temperature

As pointed out by Datta^70^, if any dimension of the conductor is smaller than one of the free characteristic lengths scales: (1) the Fermi wave length of electrons, (2) the mean free path of electrons, and (3) the phase-relaxation length, the conductor will exhibit conductance quantization behavior. For a conductor with a traverse constriction of the order of the Fermi wavelength, the Schrodinger equation can be decomposed into traverse and longitudinal equations. The electron transport through the constriction becomes then a simple 1D tunneling behavior^71^. The dispersion curve of the electronic subbands can be expressed as:

3 $$E\left({p}_{z},z\right)={\epsilon }_{n}+\frac{{({hp}_{z})}^{2}}{{(2\pi )}^{2}2m}$$

where z and p_z_ are the coordinates in the longitudinal z-direction in real- and in k-space, respectively, m is the effective mass of the electron, and h is the Planck constant. It means that the dispersion curve consists of discrete parabolic bands. The quantized energy levels ε_n_ strongly depend on the thickness of the nanofilament. Thinner filaments have larger energy spacings of Δε_n_ leading to stronger spacing out of the subbands.

The current through such constriction can be written as^70^:

4 $$I=GV \,with \, G=N\cdot {G}_{o}\cdot {\sum }_{i,j}{T}_{i,j } \, and \, {G}_{o}=\frac{2{e}^{2}}{h}$$

where T_i,j_ are the transmission probabilities from incoming channel i to the outgoing channel j, where N is the total number of available channels and e denotes the elementary electronic charge. Quantized conductance described above has been confirmed in numerous instances of atomic contacts at room temperature and in absence of a magnetic field.

There exists a large body of literature reporting observation of quantization of conductance G in units of G_o_ [see Eq. (4)] in various types of nanowires and or point contacts^72–86^, including nanowires formed in RRAM cell structures^2,71,87–89^, very similar to the Cu nanofilaments addressed in this work. There are several methods of creating atomic size constrictions. One method of creating a nanowire has been achieved by stretching or thinning of metallic wires, called mechanically controlled break junction (MCBJ). Takayanagi et al.^73^ and Rodrigues et al.^77^ have observed quantized conductance in sequential steps at 300 K in gold nanowires generated by stretching until the nanowire breaks after forming a single atom chain. Lagos et al.^74^ have found the same quantum conductance effects in silver nanowires created by mechanical elongation. Another method to produce a chain of metal ions consists in using of scanning transmission microscope (STM). Here, the nanowire is formed between the metallic sample and the STM tip. As the tip is being pulled back, the nanowire gradually decreased in cross-section with conductance G behaving like a staircase function for a constant applied voltage. Such STM experiments have been conducted by Garcia et al.^75^, by Cui et al.^85^, and by Ohnishi et al.^50^ on gold nanowires, and by Costa-Kraemer et al.^76^, Mehrez et al.^81^, and Ciraci et al.^80^ on Cu and Pt nanowires. Ohnishi et al. deduced also that the interatomic distances in the thin strand of Au atoms were quite large, between 0.35 to 0.5 nm, significantly larger than the average spacing of Au atoms of 0.29 nm in a gold bulk material. Quantum conductance has also been observed in numerous ReRAM cell structures. Nishi et al.^87^ observed quantized conduction during the final stages of the forming process of a conductive filament in Pt/NiO/Pt structures. The CF consisted of a chain of oxygen vacancies formed in the NiO dielectric film. The same quantized conductance behavior has been observed in Au/Ta_2_O_5_/Au^83^, Pt/HfO_x_/Pt^89^, and Pt/NiO/Pt^87^ resistive memory cells. In case of Au/Ta_2_O_5_/Au^88^, the quantum conductance effects were observed not during the last stages of nanofilament formation or set operation, but during the last stages of the reset switching event, i.e. during the final stage of the filament rupturing process. In fact, the quantized conductance, has been observed also in the cells used for this work, i.e. Cu/TaO_x_/Pt cells, during the final stages of the set or forming process of the nanofilment using a very low voltage ramp, see Fig. 6 in Ref.^2^. Other methods of manufacturing of nanowires where quantized conductance has been observed include formation of metallic protrusions (silver protrusions, Terabe et al.^78^) and growth on nanowires be chemical vapor deposition, e.g.^83,84^.

**Figure 5:**
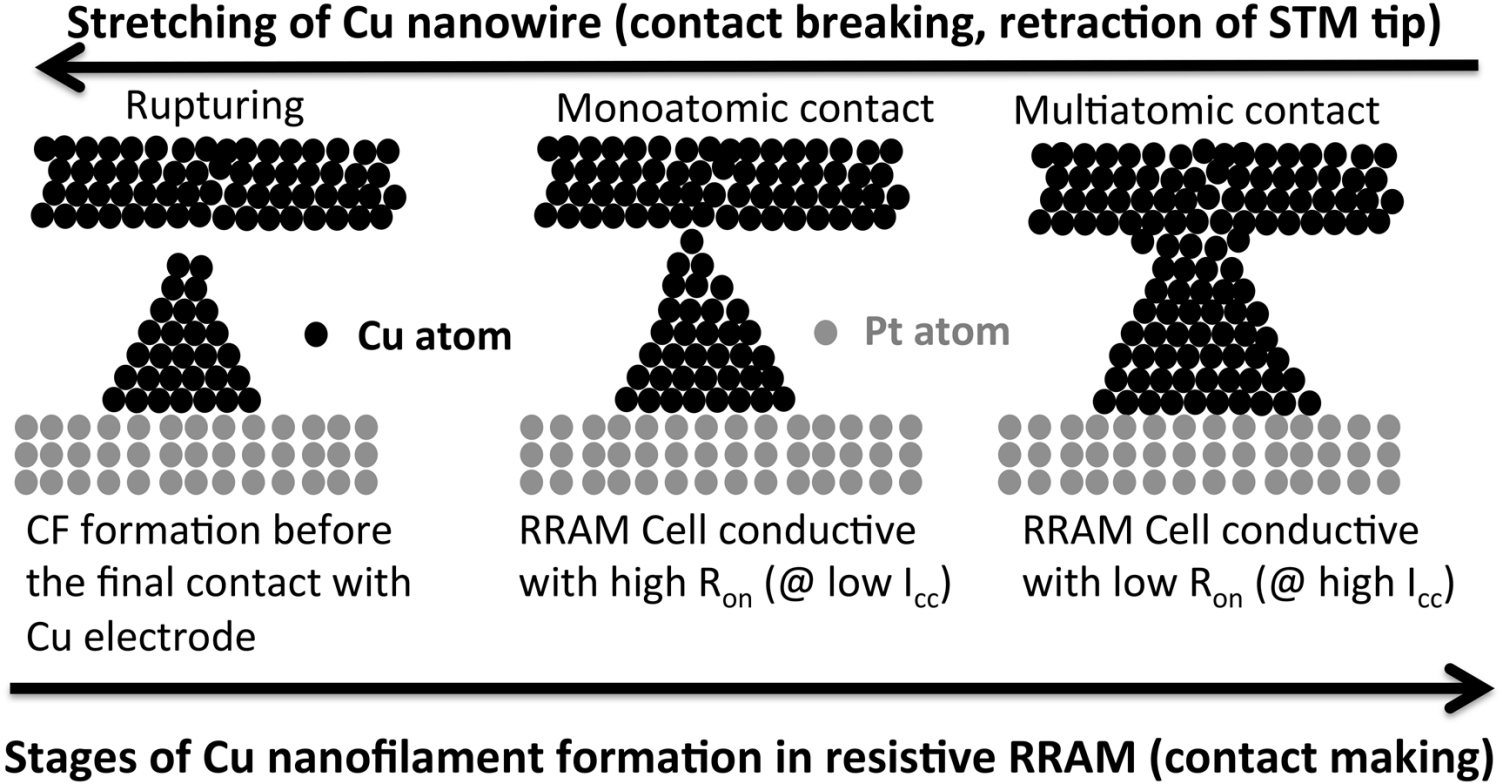
Equivalency between making a monoatomic contact by elongation of an indented Cu wire in a free space and formation of a chain of Cu atoms in the matrix of a thin dielectric (TaO_x_) in a ReRAM cell and electrochemical deposition in CuSO_4_ ion solution and a method for monoatomic contact formation using Scanning Transmission Microscope (STM). For both types of point contact fabrication, quantization of conductance in units G_o_ = 2e^2^/h has been observed in large number of publications cited in this paper.

Thus, there is an ample evidence that, irrespective of the various manufacturing techniques to produce atomic-sized contacts and the complicated atomic structure of such contacts, as soon as the weakest point is reduced to just a few atoms or a single atom, the complexity of methods and structures is removed and the result is an observed quantization of conductance in units of G_o_. The universal features of all the experiments are summarized in Fig. 5. If one proceeds from the right to the left, the three stages shown in Fig. 5 represent sequentially the mechanical stretching or rupture or the retraction of STM tip or a separation of the electrode from a protrusion, or the rupturing of a nanofilament in a reset operation of the ReRAM structure^62,63^. Viewed in opposite direction the three stages represent the nanofilament formation as it occurs in resistive memory cells during the form or set operation or in dendritic growth in chemical solutions.

In resistive memory cells, such as in the present Cu/TaO_x_/Pt cells, the nanofilament is created by metallic ions such as Cu^+^ or by conductive oxide defects embedded in the matrix of the dielectric. It is clear that the density of Cu atoms in a conductive Cu nanofilament in TaO_x_ is much lower than in the Cu bulk material. Therefore, similarly to the Ohnishi’s finding^50^ of stretched Au nanowires, the average distance of Cu atoms in the nanofilament is considerably larger than between Cu atoms in Cu bulk. This has the consequence that the quantum conductance is not just G_o_ = 2e^2^/h but G_o_ = 2e^2^/h) × t, where t is the transmission probability associated with electron tunneling from one Cu atom to the nearest Cu atom neighbor assuming that there is a difference of chemical potential between the two atoms. Formula for the transmission probability between two Cu atoms due to tunneling is given by

5 $$t\sim \mathrm{exp}\left(-2pa\right) \, with \, p=\sqrt{\frac{2m{U}_{o}}{{\left(h/2\pi \right)}^{2}}}$$

Here, direct tunneling through a rectangular barrier of height *U*_*o*_ and of width *a* is being assumed, for the sake of simplicity of the analytical structure of the formula for the tunneling probability. The distance between Cu atoms in the filament in TaO_x_ material matrix, typically *a* ≈ 0.3–0.5 nm as discussed before. When the filament is heated up, the vibrations of Cu atoms of the nanofilament are bound to increase. A simple model for vibrating atoms is the harmonic oscillator approximation. The potential energy of an oscillator is given by (1/2)k_s_x^2^ ~ (1/2)kT per degree of freedom. Here, k_s_ is the spring constant which can be extracted from the interatomic potential, k is the Boltzmann constant, and x the elongation of the spring. Therefore the square of the amplitude *a* of the harmonic oscillations is proportional to absolute temperature T, i.e. *a*^2^ ~ T. At room temperature the oscillations are moderate and the average electron tunneling can proceed more or less at a noticeable rate. However, at an elevated temperature such as 700 °C or 900 °C, corresponding to 972 K or 1172 K, the oscillation amplitudes of each individual Cu atom may increase up to a factor of $\sqrt{972/300}=1.8$ or $\sqrt{1172/300} =2.0$, respectively. This number is a conservative estimate as both Cu atoms participating in the tunneling event of an electron are randomly oscillating. As every of the Cu atoms may vibrate in a statistically random way, the average distance between neighboring Cu atoms at 1172 K can increase by up to 2.0 × $\sqrt{2}$. An increase of tunneling distance by a factor of 2 or more, will decrease exponentially the tunneling probability t given in Eq. (5) by several orders of magnitude, depending on the magnitude of the barrier height, *U*_*o*_.

**Figure 6:**
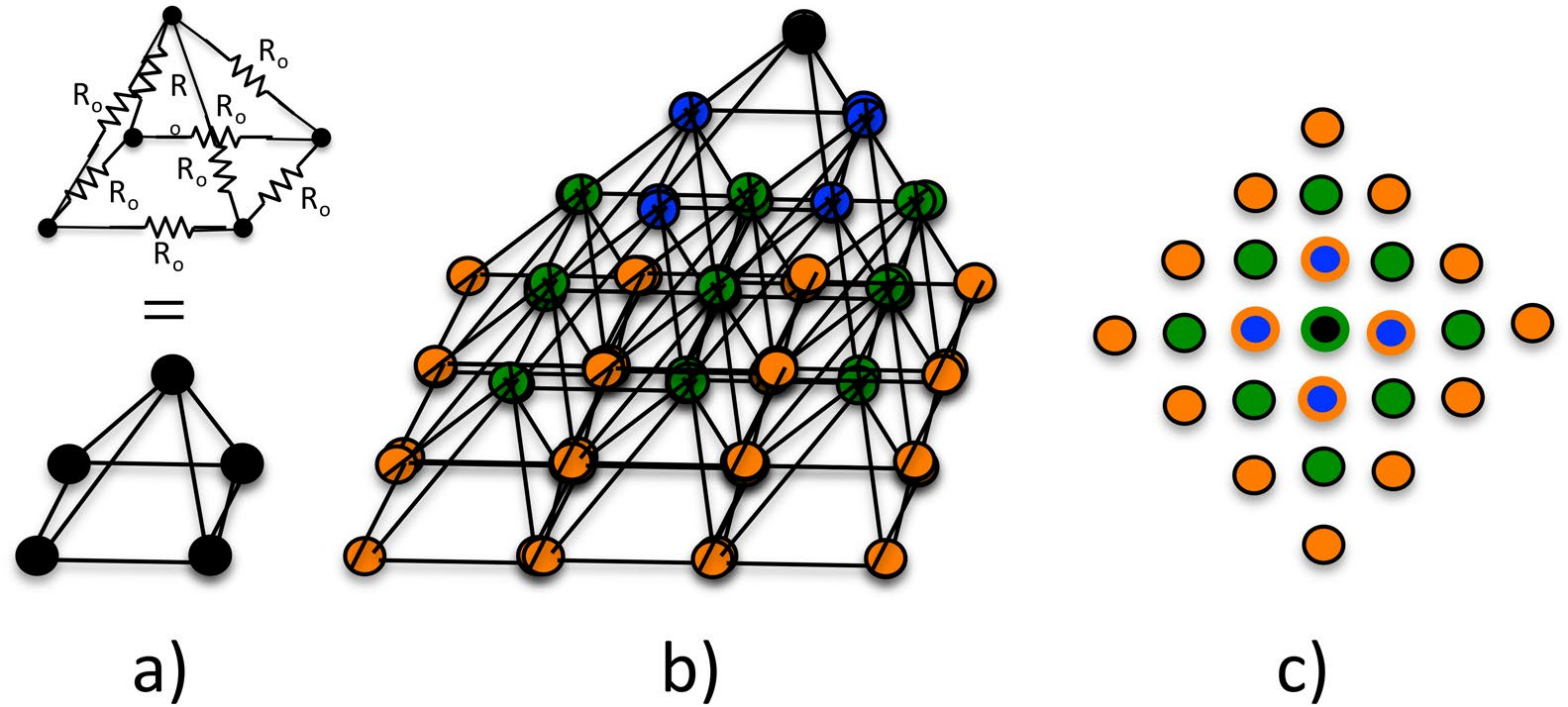
(**a**) Every Cu atom is connected to four Cu atoms in the next subjacent layer. Each connection between the Cu atoms corresponds to a unit resistance of R_o_ = 1/G_o_. (**b**) Pyramidal 3D resistor network with one atom at the top and three subjacent layers shown. (**c**) Projection of the pyramid onto the plane of the base of the pyramid. The first layer consists of one atom (black) the 4th layer consists of 16 atoms (orange). In general, nth layer consists of n^2^ atoms.

Based on the sparsity and the limited number of the Cu atoms in a Cu filament, we are constructing a 3D resistance network in the shape of a pyramid as shown in Fig. 6b as a model of the resistance of the Cu nanofilament^90,91^. The total resistance of the pyramidal 3D network is denoted by R_CF_. The vertex of the pyramid at the Cu electrode consists in an extreme case shown in Fig. 4a of a single Cu atom. One atomic layer below the vertex Cu atom is connected to four other Cu atoms (Fig. 6a). Those four atoms are connected in turn to nine Cu atoms in an atomic layer below such as each Cu atom above is connected to four Cu atoms below. Since some of the Cu atom in the third layer below are shared by several Cu atoms in the second layer, we don’t have 16 by only 9 atoms in the third layer.

As the number of Cu layers increases the base of the pyramid base widens and its height increases (Fig. 6b) until the interface of the Pt electrode has been reached. The crucial assumption made here is that each connection between two neighboring Cu atoms is characterized by quantum resistance R_o_ given as the inverse of the quantum conductance G_o_, i.e. R_o_ = 1/G_o_, where G_o_^70,80^ is given by the Landauer G_o_ = (2e^2^/h) × t,^92^ see also Eq. (4).

**Figure 7:**
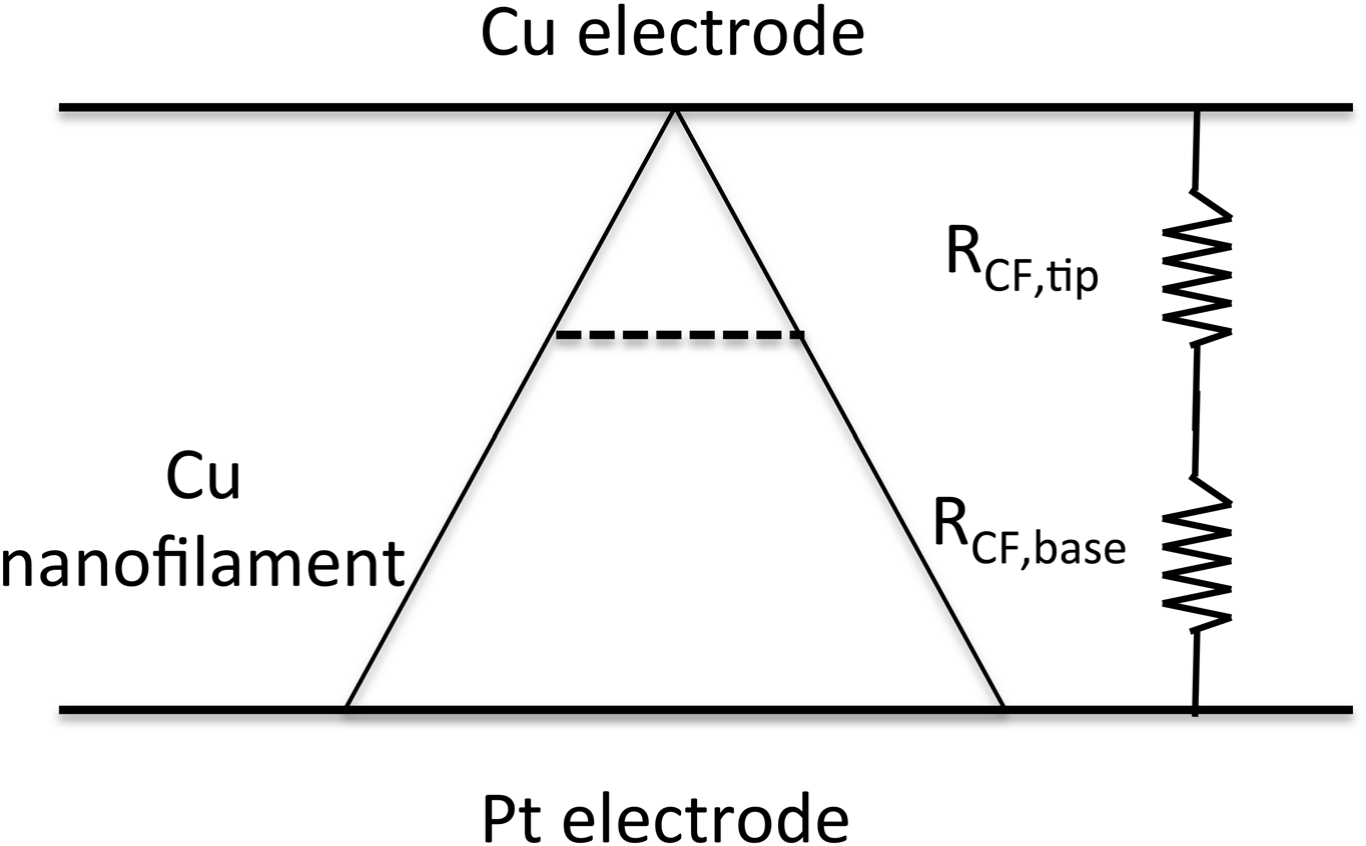
The total resistance R_CF_ of the cone or pyramidal shaped nanofilament consists of the sum of the tip and base segments: R_CF_ = R_CF,tip_ + R_CF,base_. Because of the atomic construction at the tip R_CF,tip_ >  > R_CF,base_.

Such identification of the elementary electron tunneling events with the quantum conduction is justified when the diameter of a lateral cross section of the pyramid is of the order of the Fermi wavelength. Since the electron density of electrons of a Cu filament in TaO_x_ is much smaller than the electron density of bulk Cu one can expect that the Fermi wavelength will be larger than that of bulk copper of 0.46 nm. Thus we expect the quantization effects to take place in the first few atom layers at the tip of the pyramid. Whereas at the base of the pyramid with its width hundred times larger than the Fermi wavelength, the quantization effects should be absent. Thus the resistive model in unit resistor of 1/G_o_ could apply only to the upper part of the pyramid whose resistance R_CF,tip_ constitutes the bulk of the resistance of the 3D network, i.e. R_CF,tip_ ≈ R_CF_. The broad lower part of the pyramid with resistance R_CF,base_, with R_CF,tip_ + R_CF,base_ = R_CF_ could be treated as a mesoscopic scale resistor with no quantization effects as indicated in Fig. 7.

Regardless of the nature of the two parts of resistances and because of the symmetry of the configuration, each horizontal layer can be regarded as equipotential surface and the horizontal connections between R_o_ resistors can be ignored, since no current is flowing along any horizontal cross-section of the pyramid. A straightforward algebra shows (see Fig. 6c) that the nth layer of the pyramid will have n^2^ atoms.

In terms of the 3D pyramidal network of unit resistors R_o_ as a model for the resistance of the filament, the total resistance R_CF_ of the with one atom at its vertex can be shown to be:

6 $${R}_{CF}={R}_{o}\left(1+\frac{1}{4}\sum_{n=1}^{N}\frac{1}{{n}^{2}} \right)<{R}_{o}\left(1+\frac{{\pi }^{2}}{24}\right)$$

Since the infinite sum over 1/n^2^ converges to π^2^/6, such a pyramid even of an infinite height would have a finite resistance as shown in Eq. (6). Assuming an equidistance between the Cu atom layers in the filament of 0.5 nm and given the thickness of TaO_x_ layer of 25 nm the numbers of layers would be N = 50. When the number of Cu atoms at the surface is 4 then the total resistance becomes smaller, ${\mathrm{R}}_{CF}={\mathrm{R}}_{o}\frac{1}{4}\sum_{n=1}^{51}\frac{1}{{n}^{2}}$. For 9 atoms at the surface one obtains ${\mathrm{R}}_{CF}={\mathrm{R}}_{o}\frac{1}{4}\sum_{n=2}^{52}\frac{1}{{n}^{2}}$, and for 16 atoms ${\mathrm{R}}_{CF}={\mathrm{R}}_{o}\frac{1}{4}\sum_{n=3}^{53}\frac{1}{{n}^{2}}$, and so on. These cases represent the instances of multi-atomic contact of the Cu filament with the Cu electrode interface. Table 1 shows the resistance values of the 3D resistor network as a function of the number of surface atoms forming the contact with the Cu electrode. The resistance values of the 3D resistor model are very realistic resistance estimates for the Cu filament resistance values observed experimentally in Cu/TaO_x_/Pt cells.

**Table 1:** Resistance R_CF_ of the Cu nanofilament according to the pyramidal 3D resistor network of 50 layers shown in Fig. 6 as a function of the number of top Cu atoms making contact with the Cu electrode.

# Surf. atoms	1	4	9	16	25
CF R_CF_ [Ω]	18,200	5293	2065	1278	917

From Eq. (6) the resistance of the entire 3D resistor network R_net_ is given by

7 $${R}_{net}\approx {R}_{on}={F}_{geo}\cdot {R}_{o}={F}_{geo}\frac{h}{2{e}^{2}\cdot t}\sim \mathrm{exp}\left(C\sqrt{T}\right)$$

where F_geo_ is a dimensionless factor accounting for the geometry and internal Cu atom connectivity of the 3D resistor network of identical resistors R_o_ and C = $p\times \sqrt{k/{k}_{s}}$. For example, F_geo_ for a truncated pyramid with four Cu atoms at the top is F_geo_ ≈ 0.4. Combining Eqs. (5) with (6) it can be seen that the network equivalent resistance of electron transmission events will depend exponentially on the square root of the local temperature T, as indicated in Eq. (7). In reality, this dependence may be even stronger than the oscillator amplitude since the interatomic distances may increase even more at higher temperatures due to the anharmonicity of the interatomic potential^93^, but still before diffusion jump processes of the Cu atoms may be setting in.

## Conclusions

In conclusion, we have demonstrated that a remotely heated Cu metallic filament, consisting of typically of 20 k–40 k Cu atoms, exhibits an abrupt transient decrease of resistance before returning to its original value of resistance before the heating of the filament. When the filament is allowed to cool off, the resistance of the filament reverts spontaneously in many cases to the original initial resistance value set during the preset operation. In other cases, the original resistance is not restored fully to the original value R_on_, but to somewhat higher value. The sharp transient increase of resistance is being caused by the temporal spike of local temperature. A moderate heating is needed to observe the transient resistance effect. *Moderate* heat in this context, means a sufficient amount of heat to cause the Cu atoms of the nanofilament to vibrate but not strong enough to allow outright Cu out-diffusion that would weaken the filament structurally and cause irredeemable increase of resistance. As long this condition is met, the filament is structurally unaffected by the transient heating and the temporary increase of resistance can be attributed to an increased average distance between Cu atoms oscillating around fixed equilibrium positions where the basic electron transmission mechanism is given by the electron tunneling from Cu atom to Cu atom giving rise to quantized conductance behavior. A 3D resistor circuit has been proposed constructed of resistors with a unit quantum resistance R_o_ = h/(2e^2^ × t) where t is the electron tunneling probability from Cu atom to Cu atom. This tunneling probability depends exponentially on the local temperature T of the filament and explains the restoration of the initial resistance of the conductive nanofilament after the nanofilament has cooled off.

## Supplementary Information

Supplementary Table.
