# Supplementary material for: Electron tunneling between vibrating atoms in a copper nano-filament
Source: Sci Rep. 2021 Apr 1;11:7413. doi: 10.1038/s41598-021-86603-6 (PMC8016960; doi:10.1038/s41598-021-86603-6)
Supplement: Supplementary file 1 — Supplementary Table. [file 41598_2021_86603_MOESM1_ESM.docx]

**TABLE II**

Resistance R_CF_ of the Cu nanofilament according to the pyramidal 3D resistor network of 50 layers as a function of the number of top Cu atoms making contact with the Cu electrode.

| **# surf. atoms** | **1** | **4** | **9** | **16** | **25** |
| --- | --- | --- | --- | --- | --- |
| **CF R_CF_ [Ω]** | **18,200** | **5,293** | **2,065** | **1,278** | **917** |
